# Alteration of Effective Connectivity in the Default Mode Network of Autism After an Intervention

**DOI:** 10.3389/fnins.2021.796437

**Published:** 2021-12-22

**Authors:** Han Yu, Hang Qu, Aiguo Chen, Yifan Du, Zhimei Liu, Wei Wang

**Affiliations:** ^1^Department of Radiology, Medical Imaging Center, The Affiliated Hospital of Yangzhou University, Yangzhou University, Yangzhou, China; ^2^College of Physical Education, Yangzhou University, Yangzhou, China

**Keywords:** default mode network, social communication, dynamic causal modeling (DCM), effective connectivity, exercise intervention

## Abstract

Neuroimaging has revealed numerous atypical functional connectivity of default mode network (DMN) dedicated to social communications (SC) in autism spectrum disorder (ASD), yet their nature and directionality remain unclear. Here, preschoolers with autism received physical intervention from a 12-week mini-basketball training program (12W-MBTP). Therefore, the directionality and nature of regional interactions within the DMN after the intervention are evaluated while assessing the impact of an intervention on SC. Based on the results of independent component analysis (ICA), we applied spectral dynamic causal modeling (DCM) for participants aged 3–6 years (experimental group, *N* = 17, control group, *N* = 14) to characterize the longitudinal changes following intervention in intrinsic and extrinsic effective connectivity (EC) between core regions of the DMN. Then, we analyzed the correlation between the changes in EC and SRS-2 scores to establish symptom-based validation. We found that after the 12W-MBTP intervention, the SRS-2 score of preschoolers with ASD in the experimental group was decreased. Concurrently, the inhibitory directional connections were observed between the core regions of the DMN, including increased self-inhibition in the medial prefrontal cortex (mPFC), and the changes of EC in mPFC were significantly correlated with change in the social responsiveness scale-2 (SRS-2) score. These new findings shed light on DMN as a potential intervention target, as the inhibitory information transmission between its core regions may play a positive role in improving SC behavior in preschoolers with ASD, which may be a reliable neuroimaging biomarker for future studies.

**Clinical Trial Registration:** This study registered with the Chinese Clinical Trial Registry (ChiCTR1900024973) on August 05, 2019.

## Introduction

Autism spectrum disorder (ASD) is a complex neurodevelopmental disability (Lord and Bishop, 2015) that emerges in the early stages of life, often severely impairs daily life functions, and is generally associated with life-long disability (Howlin et al., 2004). The prevalence of ASD has currently risen to one in 54 children and ASD was 4.3 times as prevalent among boys as among girls (Maenner et al., 2020). ASD is primarily manifested as persistent impairments in social communications (SC) and the presence of restricted, repetitive patterns of behaviors, interests, or activities. Despite heterogeneity in behavioral manifestations across sensory and other domains, impaired SC is a core feature of autism (Pelphrey et al., 2011; Xiao et al., 2021).

In general, SC deficits in individuals with ASD often result in exclusion from social interactions (Bolling et al., 2011), contributing to a perception of being “lost in one’s narrow world” due to the lack of initial motivation for social communications and interactions and initiating a vicious cycle. Furthermore, the terrible suffering of individuals and families also contributes to ASD being a major public health concern (Maenner et al., 2020). Therefore, timely intervention is particularly important for the healthy development of preschoolers with ASD who are in a critical period of brain development.

Various evidence-based ASD interventions aimed at improving SC ability (Roane et al., 2016), have been applied successfully. Physical exercise interventions (being low-cost, easy-to-implement, and acceptable multimodal intervention methods) may be more suitable for children and are extensively used in the improvement of brain cognitive function (Ketcheson et al., 2018; Reinders et al., 2019). Previous studies (Tse, 2020) together with our colleagues study (Cai et al., 2020) have indicated that autism receiving various exercise interventions have different degrees of improvement in SC-related outcomes. Furthermore, for preschool children with a critical brain development period, early intervention may play a particular role in facilitating the development of the nervous system, including the formation of synapses and myelination (Khundrakpam et al., 2016). In addition, a select study suggested that the earlier age of starting intervention was a statistically significant predictor of better developmental functioning and/or diagnostic status outcome in children with ASD (Towle et al., 2020). Hence, in the current context, we have implemented the 12W-MBTP, a multipath and multimodal theoretical exercise program specially designed for children with ASD, to improve the SC deficits via improving the neurophysiological state and elevating the ability of scenarios interactions.

Of note, although a deficit called “social communication” is considered one of the most universal and specific characteristics of autism in the DSM-5 diagnostic criteria (Tager-Flusberg, 2010), there is a lack of universal consensus about the underlying neural mechanism of social disorders in ASD (Venkataraman et al., 2015). This uncertainty is due to the etiological complexity and phenotypic heterogeneity as well as environmental assimilation effects of ASD. One theory (Gallagher and Frith, 2003) attempts to explain the social disorder of ASD, that is, theory of Mind (ToM) that relates to the ability of individuals to predict the behavior of others on the basis of their own mental states (goals, feelings and beliefs) and enables the identification of others’ intentions, emotions, and self-awareness. Perhaps it is not surprising that the DMN, a core brain system for processing information about the “self” and “other” (Mars et al., 2012; Molnar-Szakacs and Uddin, 2013), has been linked to both social cognition and the mentalizing process. Analysis of the extant literature in neurotypical individuals reveals that the core nodes in DMN involved in the ToM process [e.g., medial prefrontal cortex (mPFC), posterior cingulate cortex (PCC), bilateral temporoparietal junction (TPJ)] (Hyatt et al., 2015) play distinct and interacting roles in both the monitoring of the psychological state of self and the evaluation of others (Padmanabhan et al., 2017).

Synthesizing the previous literature, a universal consensus has recently been reached that the abnormal functional connectivity between the core regions of the DMN contribute to the SC impairments in ASD (Yerys et al., 2015; Padmanabhan et al., 2017). Furthermore, there is convergent evidence that SC impairments of ASD reflect a pattern of dual impairment, that is hyper/hypo-connectivity, which results in reduced specialization, flexibility, and processing efficiency of social network (e.g., DMN) (Uddin et al., 2013).

Overall, research increasingly indicates that, as a core network for mentalizing process (Buckner et al., 2008), the atypical connectivity between key regions of the DMN (Molnar-Szakacs and Uddin, 2013) affects both social network integration and differentiation (Yerys et al., 2015), resulting in deficits in SC of ASD (Molnar-Szakacs and Uddin, 2013). Unfortunately, the nature, directional and extent of this atypical functional connectivity remain sharply debated (Uddin et al., 2015), leading to difficulties in accurately understanding the potential mechanisms and intervention targets of ASD social disorders. Moreover, to the best of our knowledge, research has rarely explored the underlying neurophysiological mechanism in ASD after an intervention. Therefore, to examine the directionality and nature of the specific influence of one region on another region, we established a DCM (Friston et al., 2003) for resting-state functional magnetic resonance imaging (fMRI) cross-spectral densities (Friston et al., 2014) among different brain regions of preschoolers with ASD based on the results of ICA. Finally, applying Pearson correlation analysis to ask whether any directed connections are related to symptom severity to establish the symptom-based validation. Compared with other methods of causality analysis, the DCM not only is more suited to disclose the causality and directed nature of the coupling between intrinsic modes of brain activity but also can effectively avoid the suspicious error caused by “lag-based” causality in Granger causality analysis (Webb et al., 2013; Zhou et al., 2018).

This original research aims to investigate the underlying neural mechanisms of exercise interventions in enhancing social communication (SC) skills for preschoolers with autism by using the spectra DCM. Therefore, we hypothesized the exercise-induced effects may alter the imbalance of local excitatory/inhibitory in the DMN feedback loop, improving the influence of secondary pathological processes caused by chronically elevated metabolic stress and relieving the symptoms of social deficits in preschoolers with ASD.

## Materials and Methods

### Study Design

This research design with a two-factor (time and group) repeated measurements was conducted between October and December 2018 in Yangzhou, China. The current research is mainly aimed at preschool (3–6 years old) with ASD to reduce the deviation caused by age development (Vander Wyk et al., 2014). Research of young children is also particularly critical for developing neuroimaging biomarkers of the disorder (Padmanabhan et al., 2017).

### Participants Selection

All participants were outpatients diagnosed by pediatricians in a tertiary hospital based on the Diagnostic and Statistical Manual of Mental Disorders 5th-edition (DSM-5) and assisted by Childhood Autism Rating Scale (CARS) (Schopler et al., 1980). Potential participants were excluded if they met any of the following criteria: (1) involvement in a structured exercise program in the past 6 months; (2) a history of substance abuse or dependence in the last 6 months; (3) co-morbid psychiatric or neurological disorders; (4) visual or auditory impairments; (5) intracranial lesions that affect image analysis; (6) exercise contraindications in medical rehabilitation; (7) contraindications for magnetic resonance imaging (MRI) scanning.

Ninety-four participants were recruited from Chuying Child Development Center and Starssailor Education Institution (Yangzhou, China). Of the initially diagnosed ASD preschoolers, participants who did not meet study criteria (*n* = 36) or declined to participate in this study (*n* = 18) were excluded so that 40 participants were finally eligible and randomly equally distributed into two groups by using simple randomization with a random number table: the experimental group (*n* = 20) and the control group (*n* = 20). Of note, participants from the two different places and their parents had no prior social interactions with one another to minimize differential expectancies. Because some subjects were unable to complete the MRI scans post-intervention (*n* = 6), and inferior images were not allowed for subsequent analysis (*n* = 3). The final data analysis: a total of 31 participants between the experimental group (*n* = 17) and the control group (*n* = 14). Before the research, explain the research including contents and precautions in detail to the parents or guardians of the participants and obtain written authorization. The study was approved by the Ethics and Human Protection Committee of the Affiliated Hospital of Yangzhou University, and complied with the ethical standards of the Helsinki declaration. Meanwhile, this study retrospectively registered with the Chinese Clinical Trial Registry (ChiCTR1900024973) on August 05, 2019.

### Behavioral Measurements

Apart from gathering information about demographics (age, sex, and body mass index) at baseline, the CARS and clinical assessment report were used to evaluate the symptom severity of ASD. The CARS is a behavior rating scale consisting of 15 items for assisting in ASD diagnosis and determine the severity. The SRS-2 (Constantino et al., 2003) is a reliable and valid 65-item teacher or parent questionnaire used to measure social disorder symptoms as they occur in natural social settings for ASD. In this work, the SRS-2 scale was filled out by parents according to the specific performance of the participants in daily life. Of note, the questionnaire must be filled in by the same person before and after intervention.

### Mini-Basketball Training Program

Besides routine behavior rehabilitation training according to the standard rehabilitation program set by the institutions, the experimental groups also received an additional 12W-MBTP, while controls maintained a consistent lifestyle and did not participate in other sports-related activities.

The MBTP was conducted by two certified physical educators, adopting the mode of combining the basic movements of a mini-basketball with sports games to design projects of varying degrees of difficulty and a collective teaching model to facilitate social interactions and communications among participants while parents were strongly inspired to accompany them throughout the curriculum. The 12W-MBTP contents (40 min × 5 sessions per week × 12 weeks, fixed time, location, and physical educators) can be simply summarized as a three-phase stage, with four small parts for each course including first 2 min classroom routine preparation, then 8 min warm-up activities, followed by a 25 min mini-basketball training program, and finally 5 min cool-down activities. More details of the intervention program, please refer to the articles published by our colleagues (Cai et al., 2020) and Supplementary Tables 1, 2. After the first phase of 12W-MBTP, participants established interest in training through some simple mini-basketball games. Although some ASD individuals lacked sufficient interest or motivation for further training, they could still complete the entire training process with the help of parents and physical educators. The average heart rate during the intervention was monitored (MD = 136.97, *SD* = 7.45) using a heart rate meter (POLAR M430) to keep the activity at a moderate intensity. Most importantly, assessments were performed after each course, and participants were removed from the study if they asked for leave for more than 2 days consecutively or more than 7 days cumulatively.

### Magnetic Resonance Imaging Acquisition Protocol

The participants were deprived of sleep with the consent of the family members or guardians and sedation with 10% chloral hydrate, a safe way for children was administered before the scan to avoid excessive head motion (Doria et al., 2010; Nordahl et al., 2016). Two groups were both scanned within 3 days before and after the intervention and were completed with the company of a guardian. If the participants began to wake up, MRI acquisition was paused.

Neuroimaging data were acquired using a 3.0 T GE scanner (GE Discovery MR750w 3.0 T, Chicago, United States) located in the Affiliated Hospital of Yangzhou University. Functional images were collected in 28 axial slices using echo-planar imaging (EPI) with T2* weighted contrast sequence sensitive to blood-oxygen-level-dependent (BOLD) contrast [voxel size: 3.5 mm × 3.5 mm × 4 mm; repetition time (TR) = 2000 ms, echo time (TE) = 30 ms; flip angle (FA) = 90°, matrix = 64 × 64, field of view (FoV) = 256 mm × 256 mm; slice thickness = 4 mm; slice gap = 1 mm]. Each fMRI session lasted 8 min and thus contained 240 volumes. High-resolution T1-weighted structural images were acquired in a sagittal orientation using a three-dimensional magnetization-prepared rapid acquisition with gradient-echo (MPRAGE) sequence (voxel size: 1 mm × 1 mm × 1 mm, no gap, TR/TE = 7.2/3.1 ms; FA = 12°; FoV = 256 mm × 256 mm; 166 slices).

### Functional Magnetic Resonance Imaging Data Processing

Conventional functional imaging preprocessing was performed using Statistical Parametric Mapping software (SPM12 version 7771) implemented in MATLAB 2013b (MathWorks, Inc., Natick, MA, United States). The initial ten volumes of each dataset were discarded, then the remaining images were corrected for differences in slice time. Motion correction was performed by aligning each participant’s time-series to the mean image, and 6 motion parameters were calculated during realignment. Participants’ data were excluded if movement in the translational or rotational planes exceeded 2.5 mm or 2.5°, respectively. Functional images were registered to the standard MRI template, unbiased age-specific structural brain atlases specially provided for the Chinese pediatric population (Zhao et al., 2019), with specific operations to be processed using advanced normalization tools (ANTs) in individual subject space and then resampled to 3 mm × 3 mm × 3 mm. Resultant images were smoothed with a Gaussian kernel full width at a half-maximum of 6 mm and high-pass filtered (128 s, ∼0.008 Hz) to remove low-frequency drifts and Higher frequencies (>0.1 Hz) were not removed, since they are known to contain meaningful information in resting-state studies (Csukly et al., 2020). These images were then used as an input for group ICA.

### Selection of Regions of Interest: Independent Component Analysis

To identify regions for subsequent DCM, ICA was performed by the Group ICA of fMRI Toolbox (GIFT). First, the optimal number of components was estimated to generate 33 independent spatial maps by using a minimum description length approach. Meanwhile, the corresponding components for each subject were calculated via a back-reconstruction step. After visually inspecting all highly relevant components, we determined the peak coordinates of each region of interest in the group level while excluding all components related to head motion, physiological noise, or cerebrospinal fluid fluctuations. Finally, subject-specific coordinates were identified as the peaks in subject-specific ICA maps within 8 mm of the group-level coordinates (Zhou et al., 2018).

### General Linear Model for Time-Series Extraction

To control motion and physiologic noise effects and correct for the influence of interindividual differences, a general linear model (GLM) containing a discrete cosine basis set and the nuisance regressors that included 6 head motion parameters and WM and CSF signals (Almgren et al., 2020), age, sex and handedness was used to model the resting-state data. The mean time series over the regions identified in ICA were extracted with the use of the principal eigenvariates (principal components) of voxels within 8 mm of the subject-specific coordinates adjusted for the confounding regressors.

### Effective Connectivity: Spectral Dynamic Causal Modeling With Parametric Empirical Bayesian

Generally, DCM analysis requires the specification of a model space (Friston et al., 2014). Due to the rarity of research on resting state in autism under intervention, we adopted an approach starting with a fully connected model. This means that all 4 identified ROIs based on ICA were connected, generating a total of 16 connectivity parameters, including inhibition of self-connections. We take advantage of the latest developments in the use of spectral DCM to model endogenous activities in the framework of parametric empirical Bayesian (PEB) (Friston et al., 2016) analysis to inform whether increases or decreases in extrinsic and intrinsic connections were present within each region at the group level. After the model specification, the full model was estimated and inverted for each subject with a hierarchical empirical Bayesian inversion, which allowed the variability in an individual subject’s connection strengths to influence the second-level analysis, thereby eliminating the between-subject degree of variability. As opposed to classical inference such as ANOVA, PEB analysis takes not only the means but also the uncertainty of individual connection strengths into account, which means that participants with more uncertain parameter estimates will be down-weighted, while participants with more precise estimates receive greater influence.

After performing the first-level analysis, we created a second-level analysis by specifying a design matrix with four regressors, including the effect of time interacting with the group that best described the effects of the intervention of the 12W-MBTP.

To finally utilize the model, we used Bayesian model reduction (BMR) (Friston et al., 2016), an automatic search method to quickly prune parameters from the second-level PEB model that do not contribute to the model evidence, to infer connections best describing the interaction effect (group × time). Bayesian model averaging (BMA) was performed for the PEB models after obtaining the probability for all possible PEB models (nested and full) separately from BMR and weighted by their model evidence rather than choosing one final model. The parameters best describing interaction effects are reported not as *p*-values but instead in terms of posterior probability values (PPs), so connections that survived a non-zero criterion with posterior probability PPs > 0.95 are considered significant.

### Correlation Analysis

Finally, we asked whether any directed connections are related to symptom severity to establish the symptom-based validation. Following our hypothesis, we then analyzed the correlation between the changes in EC and SRS-2 scores. The alterations here refer to post-intervention minus the baseline level unless otherwise specified. The analysis of the correlation was limited to the final model following second-level analysis by PEB. If a correlation was found between them, then the correlations between the connectivity strength and the items of the given SRS-2 sub-score were also analyzed.

### Statistical Analysis

The demographic analysis were performed using SPSS Version 21.0 (IBM, Armonk, NY, United States), with two-tailed independent sample *t*-tests for continuous variables and χ^2^ tests for categorical variables. We conducted two-factor (time and group) repeated measurements ANOVA analysis to evaluate the positive role of the 12W-MBTP in SC on preschoolers with ASD. Once we found significant group × time interaction effects, *post hoc* tests were performed. We calculated the mean and standard deviations of all variables and adopted the traditional cutoff of *p* < 0.05 to determine significance. The Bonferroni correction for multiple testing was applied in Pearson correlation analysis, resulting in a corrected α-value of (0.05/5 = 0.01).

## Results

### Demographic Analysis

Demographic characteristics including sex, age, and body mass index do not differ significantly between the two groups. CARS and SRS-2 scores at baseline revealed no significant differences across groups, with *p* = 0.722 and *p* = 0.396, respectively (see Table 1 for more details).

**TABLE 1:** Demographic and clinical characteristics of the participants.

Characteristics	Control group	Experimental group	*p*-value
Number	14	17	NaN
Sex (M/F)	13/2	15/2	1.000
Age (years)	4.75 ± 0.62	4.89 ± 0.80	0.520
BMI (height/weight^2^)	15.96 ± 1.85	15.68 ± 1.10	0.609
CARS (baseline)	40.50 ± 4.65	39.76 ± 6.38	0.722
SRS-2 *T*-score (baseline)	86.07 ± 20.55	93.94 ± 28.56	0.396

*BMI, body mass index; CARS, Childhood Autism Rating Scale; M/F, male/female; SRS-2, Social Responsiveness Scale – Second Edition.*

### Social Communication Performance

**FIGURE 1:**
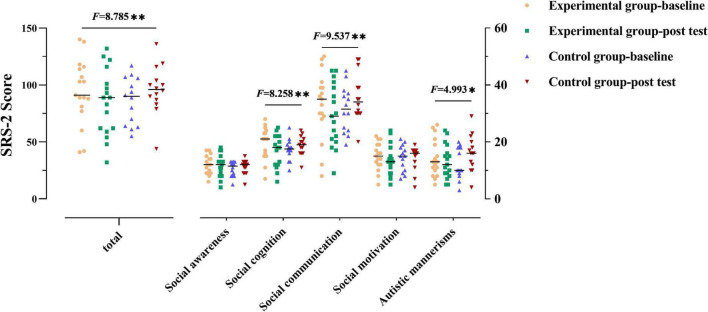
Social communication performance of the two groups. The performance for social communication symptoms of the participants in two groups before and after the intervention. The higher the score, the more severe the deficits in social communication. Numbers presented are *F* statistics representing interaction effects of groups over time. * and ^**^ means *p* < 0.05 and *p* < 0.01, respectively.

For the SRS-2 total score (as shown in Figure 1 and Table 2), not surprisingly, a group × time interaction effect was observed [*F*_(1_,_29)_ = 8.785, *p* = 0.006]. Follow-up simple effect analysis indicated that the SRS-2 total score of the experimental group at the post-test were significantly lower relative to baseline [*F*_(1_,_29)_ = 4.586, *p* = 0.041], whereas such a positive effect was not found in the control group [*F*_(1_,_29)_ = 4.240, *p* = 0.049]. Notably, a greater SRS-2 total score indicates worse SC performance.

**TABLE 2:** Analysis of two groups for social communication variables (mean ± standard deviation).

Characteristics	Control group (*n* = 15)		Experimental group (*n* = 17)		*F*
	Baseline	Post-test	Baseline	Post-test	
SRS-2 *T* score	86.07 ± 20.548	95.86 ± 21.343	93.94 ± 28.575	84.71 ± 29.146	8.785**
Social awareness	10.50 ± 2.534	11.14 ± 2.476	12.00 ± 3.240	11.65 ± 3.904	1.461
Social cognition	17.29 ± 3.688	18.79 ± 3.378	19.71 ± 5.520	17.18 ± 5.670	8.258**
Social communication	31.43 ± 8.055	35.50 ± 8.364	33.06 ± 11.798	29.41 ± 10.932	9.537**
Social motivation	14.36 ± 4.551	14.29 ± 4.232	14.94 ± 4.723	13.41 ± 5.363	0.972
Autistic mannerisms	12.50 ± 5.488	16.14 ± 6.298	14.24 ± 6.524	13.06 ± 5.910	4.993*

F statistics representing tests of interaction effect of group by time. ** and * means p < 0.01, p < 0.05, respectively.

Group × time interaction effect was only observed in the three subscales (social cognition, social communication, and autistic mannerisms: *F*_(1_,_29)_ = 8.258, 9.537, 4.993, *p* = 0.008, 0.004, 0.033, respectively]. Follow-up simple effect analysis results for the subscales of SRS-2 as follows: (1) the pre-test social cognition score was significantly higher than that at post-test in the experimental group [*F*_(1_,_29)_ = 7.206, *p* = 0.012], where no significant change from the post to pre-test was observed in the control group; (2) the post-test social communication score was significantly lower than that at baseline in the experimental group [*F*_(1_,_29)_ = 4.715, *p* = 0.038], whereas a higher score was observed in the control group from the post-test to pre-test [*F*_(1_,_29)_ = 4.839, *p* = 0.036; higher scores indicate severe symptoms]; (3) there was no significant difference between baseline and post-test in the experimental group [*F*_(1_,_29)_ = 0.659, *p* > 0.05] in terms of autistic mannerisms, whereas the post-test score was significantly higher than that at baseline in the control group [*F*_(1_,_29)_ = 5.202, *p* = 0.03]. All the above simple effect analysis uses Bonferroni correction to correct multiple comparisons.

### The Dynamic Causal Modeling Analysis and Effective Connectivity of Default Mode Network

The ICA was successfully selected four ROIs: mPFC (3,49,0), PCC (0,−52,23), LTPJ (−48,−63,10), and RTPJ (50,−55,11) in the DMN that specifically engaged by in the ToM process, which were selected as nodes for subsequent DCM.

The EC parameters of each specific effect obtained following group-level analysis were listed in Table 3. Please refer to Supplementary Table 3 for the specific connection strength of each connection of participants before and after the exercise intervention. Note that self-connection is the log of scaling parameters that multiply up or down the default value −0.5 Hz such that the positive self-connection values represent increased self-inhibition relative to the prior (Zeidman et al., 2019).

**TABLE 3:** Strength of effective connectivity in each specific effect.

Source	Target	Strength	PPs
**Effect of the interacions**			
mPFC	mPFC	0.138	1
mPFC	RTPJ	0.042	0.6406
PCC	RTPJ	–0.051	1
LTPJ	PCC	–0.066	1
**Effect of the group**			
mPFC	mPFC	0.212	1
mPFC	PCC	–0.177	1
mPFC	RTPJ	0.036	0.5782
PCC	LTPJ	–0.077	1
RTPJ	mPFC	–0.149	1
RTPJ	LTPJ	–0.034	0.5438
RTPJ	RTPJ	0.067	0.6775
**Effect of the time**			
mPFC	PCC	–0.141	1
mPFC	LTPJ	–0.11	1
mPFC	RTPJ	–0.069	1
PCC	RTPJ	–0.028	0.616
LTPJ	PCC	–0.073	1
RTPJ	mPFC	0.05	0.6658
RTPJ	PCC	0.14	1
RTPJ	LTPJ	–0.048	0.6734

*The self-connection with the same source and target is the log of scaling parameters that multiply up or down the default value −0.5Hz. PPs, posterior probability values.*

**FIGURE 2:**
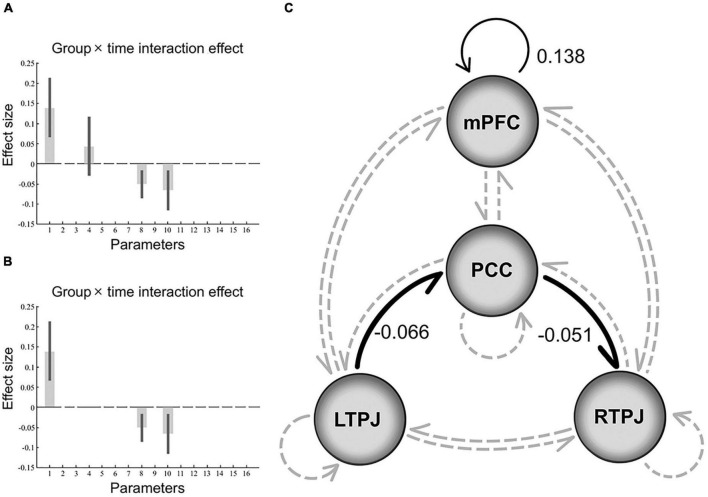
Results of the dynamic causal modeling in the group × time interaction. Panels **(A,B)** shows the effect size of each parameter in the final model that represents the interaction effect, after Bayesian model averaging, the corresponding DCM parameters with no threshold (PPs ≥ 0, graph **A**), and parameters that survived a non-zero criterion with a PPs > 0.95 (graph **B**). The black error bars are 90% credible intervals derived from the posterior variance of each parameter. Panel **(C)** depicts a schematic diagram representing the final model that includes significant negative (inhibitory) extrinsic between-regions connections in bold black line and positive intrinsic self-connections in thin black line, while non-significant connections in gray dotted line. Note that the self-connection parameter is the log of scaling parameters with no units that scale up or down the default value –0.5 Hz. So, such the positive self-connectivity values indicate increased self-inhibition than the default. PCC, posterior cingulate cortex; mPFC, medial prefrontal cortex; TPJ, temporoparietal junction.

For the effect of group × time interaction, parameters best describing the effect included the inhibitory directional connections mediated by PCC between bilateral TPJ (from left to right). Besides, the positive self-connection values observed in mPFC that showed no overall mean effect (PPs = 0) were instead the best discriminative parameters of an interaction effect, which can be explained as the size of the positive effect means an increase in self-inhibition (all PPs > 0.95, Figure 2).

### Correlations Between Effective Connectivity and SRS-2 Scores

**FIGURE 3:**
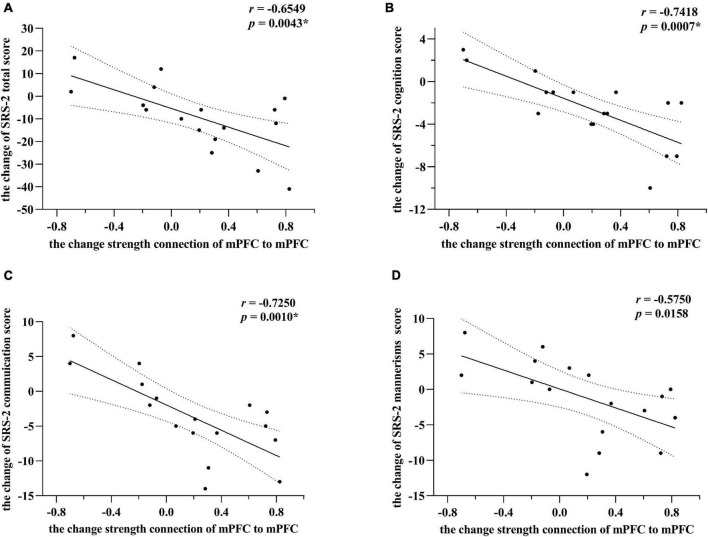
Correlations between changes of effective connectivity and SRS-2 scores in the experimental group. Panel **(A)** indicates alterations of SRS-2 total scores correlate strongest with changes in self-connection in mPFC. The alterations here mainly refer to post-intervention minus the baseline level. *Post hoc* tests showed that among SRS-2 items, social cognition and social communication scores alterations correlated significantly with changes in self-connection of mPFC in panels **(B,C)**, respectively. Panel **(D)** showed that the correlation between the alterations of autistic mannerisms scores and changes in self-connection of mPFC did not survive the correction for multiple tests. However, * means passed the multiple comparison correction. Linear regression lines and 95% confidence intervals were obtained with linear models, and statistical results are based on Pearson correlation analysis. All the results point toward that the increasing inhibitory self-connection in mPFC can predict improvement of social communications for preschool with ASD.

As illustrated in Figure 3, for the experimental group, the changes in the strength of the self-connection in mPFC correlated significantly with a change in the SRS-2 total score (Pearson *r* = −0.6549, *n* = 17, *p* = 0.0043). To further explore these results, the correlation between each of the five sub-items of the SRS-2 and self-connectivity in the mPFC was analyzed. Changes in EC of mPFC correlated significantly with the changes in social cognition and social communication sub-scores (Pearson *r* = −0.7418 and −0.7250, respectively; *n* = 17; *p* = 0.0007 and 0.0010, respectively), while the correlation with autistic mannerisms (Pearson *r* = −0.5750, *n* = 17, *p* = 0.0158) sub-score did not survive the correction for multiple tests. Unquestionably, there was no significant correlation between changes of EC strength (in the final model) and SRS-2 total scores in the control group, p value was 0.3011, 0.6348, and 0.5344, respectively.

## Discussion

Since the absence of assessment about the influence of one region to another region in functional connectivity analysis, it is impossible to reasonably explain the information exchange between brain regions in the context of physical exercise intervention. Therefore, this research aims to investigate the underlying neural mechanisms of exercise interventions in enhancing social communication (SC) skills for preschoolers with autism by using the spectra DCM in the framework of Parametric Empirical Bayesian to characterize the longitudinal changes in intrinsic and extrinsic effective connectivity in DMN core regions. Following the exercise intervention, we discovered noteworthy inhibitory directed connections in the default mode network in the experimental group, which predicted an improvement in SC skills.

Differently, previous research focused more on assessing the changes in the effective connectivity of ASD during various task states about psychology, but rarely assessed the impact of endogenous neuron fluctuations under the resting state. However, earlier effective connectivity study by multivariate Granger causality analysis with blind deconvolution have also indicated the impaired mPFC pathway and its association with SC deficits in children with ASD, which is consistent with our findings using resting state data (Li et al., 2021).

The mPFC, as the core region of the social brain network (Bzdok et al., 2013), is engaged in inferring others’ mental states, and has consistently been shown to have an abnormal overgrowth in young children with ASD from about the age of 2–5 years (Carper and Courchesne, 2005; Libero et al., 2019), with its dysfunction being closely related to the SC deficits in autism (Padmanabhan et al., 2017). Interestingly, concerning the abnormal activation of mPFC and hyper-connectivity of the intrinsic or task-related between mPFC and other regions, previous reports (Smith et al., 2013; White et al., 2014) assumed that such aberrant results may reflect a compensatory response (thus not necessarily representing an improvement of the performance of ToM) or misuse of cognitive reserve. This abnormal activation or hyper-connectivity may even be a state of underlying neuropathology or overload, which may be indicative of impending cognitive decline.

Therefore, the final model of interaction effect found strong evidence of greater self-inhibition in mPFC, indicating a decrease in the gain or excitability of this structure (Zeidman et al., 2019). Similarly, in the final model of group and time effect, mPFC also exhibited an inhibitory effect on other ROIs. To our knowledge, the DCM parameters about intrinsic self-connections lend self-inhibitory properties to regions to preclude any run-away excitation (Csukly et al., 2020), as possibly mediated by increase in postsynaptic inhibition. Specifically, it may cause mPFC to enhance the inhibition of output activity transmitted to others, which leads to changes in the connectivity of the brain circuits.

Interestingly, Prat et al. (2016) found that the non-selective replication of signals in the basal ganglia of individuals with ASD resulting in the information (including some that is irrelevant to the task at hand) overload of the prefrontal cortex. So, such an increase in mPFC self-connection inhibition as mediated by increasing postsynaptic inhibition in our results predicates the improvement of SC.

Furthermore, our findings are also consistent with a prominent neurobiological theory that an excitatory/inhibitory (E/I) imbalance (Rubenstein and Merzenich, 2003; Vattikuti and Chow, 2010; Yizhar et al., 2011) in local neural circuits alters local and global brain signals. These abnormal patterns are critically likely to affect brain function, especially if they occur in highly interconnected hubs, such as mPFC (Padmanabhan et al., 2017), leading to an obstacle in social cognition. A rodent study showed that the elevated *E*/*I* ratio in mPFC is related to an SC impaired, and this effect can be improved by increasing the inhibitory function (Yizhar et al., 2011; Zhang et al., 2020). Taken together, similar to our results, enhancing the self-inhibition of mPFC after the 12W-MBTP intervention exerts a positive effect on the SC of preschoolers with ASD.

From the significant negative correlations between changes in EC and SRS-2 score (as illustrated in Figure 3), greater mPFC self-inhibition changes (including the conversion from disinhibition to inhibition, the decrease of disinhibition and the increase of inhibition) indicated more extreme significant improvement in SC. This exciting result also confirms our previous conjectures and provides preliminary evidence for possible neural correlates of SC. That is, as a potential target for physical exercise intervention, the increased mPFC self-connection inhibition can predict better improvement of SC.

Another finding supporting the theory is that in the final model of interaction effect, the inhibitory directional connection from left TPJ to right TPJ was mediated by PCC. This result may seem a counterintuitive finding at first, and it does reflect profound and complex changes in these brain regions and underscores their importance for restoring and maintaining SC among preschoolers with ASD. Recently a new perspective proposes that there may be atypical maturational trajectories in ASD (Nomi and Uddin, 2015; Uddin et al., 2015; Muller and Fishman, 2018), that is, early hyperconnectivity followed by decreased connectivity in adulthood (Assaf et al., 2010), which may be caused by the imbalance of *E*/*I* ratio during the development of neural systems. One likely reason is the gene-, receptor-, and enzyme-level deficits in inhibitory signaling pathways involving gamma-aminobutyric acid (GABA) (Baroncelli et al., 2011; Pizzarelli and Cherubini, 2011). Not surprisingly, enhance GABAergic signaling can bridge the gaps and obtain better social cognition (Han et al., 2014; Zhang et al., 2020).

Taken together, we speculate that the inhibitory information transmission between bilateral TPJ caused by exercise intervention altered the imbalance of local E/I, improving the influence of secondary pathological processes caused by chronically elevated metabolic stress (Hillary and Grafman, 2017). Such a shocking effect can rehabilitate the homeostatic aspects of the DMN on the nervous system through increasing postsynaptic inhibition (Laughlin and Sejnowski, 2003), predicting the improvement in SC skills for autism.

Concerning the high-level cluster in the social brain, most core regions (left TPJ instead of right) exhibited a left-favored lateralization pattern of functional connectivity (Tomasi and Volkow, 2012; Alcala-Lopez et al., 2018). Unsurprisingly, autism exhibits remarkably reduced left lateralization in connections involving regions from the DMN essential for SC and maybe predicting more severe SC deficits (Nielsen et al., 2014). Combined with results of the overall mean effect, the left-sided brain function of preschoolers with ASD regained the dominant position in the process of social cognition and undoubtedly showed better SC symptoms after the 12W-MBTP intervention.

Behaviorally, the classical role of PCC is to quickly switch between different cognitive processes to coordinating external and internal-oriented cognition (Leech et al., 2012). After the 12W-MBTP, the similar regulatory effect of PCC, namely mediates the inhibitory information transmission, changed the status of so-called network isolation (Uddin et al., 2015), which is also reflected in the drops in SRS-2 scores of experimental group participants.

We presume the impact of intervention is primarily because of the exercise-induced neuroplasticity caused by the multipath effect. Evidence-based investigations have confirmed continuous moderate-intensity aerobic exercise will increase basal peripheral brain-derived neurotrophic factor (BDNF) concentrations (Knaepen et al., 2010). BDNF is generally thought to be a key neurotrophin in supporting neuroplasticity. Not only was neuromotor activity significantly improved, but the release of endogenous neurotrophins was also related to the improvement of social cognition (Baker et al., 2010). Apart from that, the exercise-induced cholinergic effects may increase cerebral perfusion, possibly affecting the neurodynamics of the BOLD fMRI signal (Smith et al., 2010) that may reflect altered brain connections and improved network efficiency.

### Limitations

There are a few limitations of our work must be taken into consideration and addressed by future studies. The lack of ADOS or ADI-R assessment might also be a potential limitation of the study. For preschoolers with autism, it is difficult to successfully recruit more participants on the premise of successful completion of the entire intervention process and two MRI scans. On the other hand, given the male preponderance in the prevalence of autism (Maenner et al., 2020), the profiles of SC impairments in ASD likely differ between sex (Muller and Fishman, 2018). Although there is a good sex matching between groups in our research, the existence of a high male/female ratio limits the applicability of our results to all populations. Larger scale studies, especially with the same proportion of males to females, are required to replicate and validate these findings. Furthermore, analyzing the EC of complex network model (the triple network model) (Menon, 2011) interaction not only is a top priority but may also provide a brand-new perspective for understanding the potential neuropathological mechanism of patients with ASD in SC deficits.

## Conclusion

Taken together, this study provides sufficient evidence that exercise intervention can improve SC skills for preschoolers with ASD. Such inspiring results also emphasize the importance of early and timely intervention for the neurodevelopmental of ASD and improvement of core symptoms and quality of life. By applying spDCM in the framework of PEB to characterize the longitudinal changes in intrinsic and extrinsic effective connections between the DMN, we conclude that the inhibitory information transmission in DMN may be an underlying neurophysiological mechanism for improving SC behaviors among preschoolers with ASD who delay or stunt maturational processes. Finally, these findings provide further factual evidence that the directional effectivity connection between key regions of the DMN has become one of the well-validated targets for exercise intervention to improve SC behaviors, which may be a reliable neuroimaging biomarker or endophenotype in subsequent studies.

## Data Availability Statement

The original contributions presented in the study are included in the article/Supplementary Material, further inquiries can be directed to the corresponding author.

## Ethics Statement

The studies involving human participants were reviewed and approved by The Ethics and Human Protection Committee of the Affiliated Hospital of Yangzhou University. Written informed consent to participate in this study was provided by the participants’ legal guardian/next of kin.

## Author Contributions

HY and WW drafted the manuscript, performed all administrative tasks required for submission, and contributed to the identification of research topics and preparation of study design. HY and HQ took part in planning, supervision, brainstorming the manuscript, designed the study, and contributed to the statistical analysis and interpretation of results. HY, AC, YD, and ZL designed the intervention method and guided the intervention process. All authors critically revised the manuscript and approved the final version.

## Conflict of Interest

The authors declare that the research was conducted in the absence of any commercial or financial relationships that could be construed as a potential conflict of interest.

## Publisher’s Note

All claims expressed in this article are solely those of the authors and do not necessarily represent those of their affiliated organizations, or those of the publisher, the editors and the reviewers. Any product that may be evaluated in this article, or claim that may be made by its manufacturer, is not guaranteed or endorsed by the publisher.

## References

[B1] Alcala-Lopez D. Smallwood J. Jefferies E. Van Overwalle F. Vogeley K. Mars R. B. (2018). Computing the social brain connectome across systems and states. *Cereb. Cortex* 28 2207–2232. 10.1093/cercor/bhx121 28521007

[B2] Almgren H. Van de Steen F. Razi A. Friston K. Marinazzo D. (2020). The effect of global signal regression on DCM estimates of noise and effective connectivity from resting state fMRI. *Neuroimage* 208:116435. 10.1016/j.neuroimage.2019.116435 31816423 PMC7014820

[B3] Assaf M. Jagannathan K. Calhoun V. D. Miller L. Stevens M. C. Sahl R. (2010). Abnormal functional connectivity of default mode sub-networks in autism spectrum disorder patients. *Neuroimage* 53 247–256. 10.1016/j.neuroimage.2010.05.067 20621638 PMC3058935

[B4] Baker L. D. Frank L. L. Foster-Schubert K. Green P. S. Wilkinson C. W. McTiernan A. (2010). Effects of aerobic exercise on mild cognitive impairment: a controlled trial. *Arch. Neurol.* 67 71–79. 10.1001/archneurol.2009.307 20065132 PMC3056436

[B5] Baroncelli L. Braschi C. Spolidoro M. Begenisic T. Maffei L. Sale A. (2011). Brain plasticity and disease: a matter of inhibition. *Neural. Plast.* 2011:286073. 10.1155/2011/286073 21766040 PMC3134991

[B6] Bolling D. Z. Pitskel N. B. Deen B. Crowley M. J. McPartland J. C. Kaiser M. D. (2011). Enhanced neural responses to rule violation in children with autism: a comparison to social exclusion. *Dev. Cogn. Neurosci.* 1 280–294. 10.1016/j.dcn.2011.02.002 21743819 PMC3129780

[B7] Buckner R. L. Andrews-Hanna J. R. Schacter D. L. (2008). The brain’s default network: anatomy, function, and relevance to disease. *Ann. NY Acad. Sci.* 1124 1–38. 10.1196/annals.1440.011 18400922

[B8] Bzdok D. Langner R. Schilbach L. Engemann D. A. Laird A. R. Fox P. T. (2013). Segregation of the human medial prefrontal cortex in social cognition. *Front. Hum. Neurosci.* 7:232. 10.3389/fnhum.2013.00232 23755001 PMC3665907

[B9] Cai K. Yu Q. Herold F. Liu Z. Wang J. Zhu L. (2020). Mini-Basketball training program improves social communication and white matter integrity in children with autism. *Brain Sci.* 10:803. 10.3390/brainsci10110803 33142694 PMC7693206

[B10] Carper R. A. Courchesne E. (2005). Localized enlargement of the frontal cortex in early autism. *Biol. Psychiatry* 57 126–133. 10.1016/j.biopsych.2004.11.005 15652870

[B11] Constantino J. N. Davis S. A. Todd R. D. Schindler M. K. Gross M. M. Brophy S. L. (2003). Validation of a brief quantitative measure of autistic traits: comparison of the social responsiveness scale with the autism diagnostic interview-revised. *J. Autism. Dev. Disord.* 33 427–433. 10.1023/a:1025014929212 12959421

[B12] Csukly G. Szabo A. Polgar P. Farkas K. Gyebnar G. Kozak L. R. (2020). Fronto-thalamic structural and effective connectivity and delusions in schizophrenia: a combined DTI/DCM study. *Psychol. Med.* 2020:859. 10.1017/S0033291720000859 32329710 PMC8426148

[B13] Doria V. Beckmann C. F. Arichi T. Merchant N. Groppo M. Turkheimer F. E. (2010). Emergence of resting state networks in the preterm human brain. *Proc. Natl. Acad. Sci. USA* 107 20015–20020. 10.1073/pnas.1007921107 21041625 PMC2993415

[B14] Friston K. J. Harrison L. Penny W. (2003). Dynamic causal modelling. *Neuroimage* 19 1273–1302. 10.1016/s1053-8119(03)00202-7 12948688

[B15] Friston K. J. Kahan J. Biswal B. Razi A. (2014). A DCM for resting state fMRI. *Neuroimage* 94 396–407. 10.1016/j.neuroimage.2013.12.009 24345387 PMC4073651

[B16] Friston K. J. Litvak V. Oswal A. Razi A. Stephan K. E. van Wijk B. C. M. (2016). Bayesian model reduction and empirical Bayes for group (DCM) studies. *Neuroimage* 128 413–431. 10.1016/j.neuroimage.2015.11.015 26569570 PMC4767224

[B17] Gallagher H. L. Frith C. D. (2003). Functional imaging of ‘theory of mind’. *Trends Cogn. Sci.* 7 77–83. 10.1016/s1364-6613(02)00025-6 12584026

[B18] Han S. Tai C. Jones C. J. Scheuer T. Catterall W. A. (2014). Enhancement of inhibitory neurotransmission by GABAA receptors having alpha2,3-subunits ameliorates behavioral deficits in a mouse model of autism. *Neuron* 81 1282–1289. 10.1016/j.neuron.2014.01.016 24656250 PMC4079471

[B19] Hillary F. G. Grafman J. H. (2017). Injured brains and adaptive networks: the benefits and costs of hyperconnectivity. *Trends Cogn. Sci.* 21 385–401. 10.1016/j.tics.2017.03.003 28372878 PMC6664441

[B20] Howlin P. Goode S. Hutton J. Rutter M. (2004). Adult outcome for children with autism. *J. Child Psychol. Psychiatry* 45 212–229. 10.1111/j.1469-7610.2004.00215.x 14982237

[B21] Hyatt C. J. Calhoun V. D. Pearlson G. D. Assaf M. (2015). Specific default mode subnetworks support mentalizing as revealed through opposing network recruitment by social and semantic FMRI tasks. *Hum. Brain Mapp.* 36 3047–3063. 10.1002/hbm.22827 25950551 PMC6869394

[B22] Ketcheson L. Hauck J. L. Ulrich D. (2018). The levels of physical activity and motor skills in young children with and without autism spectrum disorder, aged 2-5 years. *Autism* 22 414–423. 10.1177/1362361316683889 29152992

[B23] Khundrakpam B. S. Lewis J. D. Zhao L. Chouinard-Decorte F. Evans A. C. (2016). Brain connectivity in normally developing children and adolescents. *Neuroimage* 134 192–203. 10.1016/j.neuroimage.2016.03.062 27054487

[B24] Knaepen K. Goekint M. Heyman E. M. Meeusen R. (2010). Neuroplasticity - exercise-induced response of peripheral brain-derived neurotrophic factor: a systematic review of experimental studies in human subjects. *Sports Med.* 40 765–801. 10.2165/11534530-000000000-00000 20726622

[B25] Laughlin S. B. Sejnowski T. J. (2003). Communication in neuronal networks. *Science* 301 1870–1874. 10.1126/science.1089662 14512617 PMC2930149

[B26] Leech R. Braga R. Sharp D. J. (2012). Echoes of the brain within the posterior cingulate cortex. *J. Neurosci.* 32 215–222. 10.1523/JNEUROSCI.3689-11.2012 22219283 PMC6621313

[B27] Li L. He C. Jian T. Guo X. Xiao J. Li Y. (2021). Attenuated link between the medial prefrontal cortex and the amygdala in children with autism spectrum disorder: Evidence from effective connectivity within the “social brain”. *Prog. Neuropsychopharmacol. Biol. Psychiatry* 111:110147. 10.1016/j.pnpbp.2020.110147 33096157

[B28] Libero L. E. Schaer M. Li D. D. Amaral D. G. Nordahl C. W. (2019). A longitudinal study of local gyrification index in young boys with autism spectrum disorder. *Cereb. Cortex* 29 2575–2587. 10.1093/cercor/bhy126 29850803 PMC6519847

[B29] Lord C. Bishop S. L. (2015). Recent advances in autism research as reflected in DSM-5 criteria for autism spectrum disorder. *Annu. Rev. Clin. Psychol.* 11 53–70. 10.1146/annurev-clinpsy-032814-112745 25581244

[B30] Maenner M. J. Shaw K. A. Baio J. Washington A. Patrick M. (2020). Prevalence of autism spectrum disorder among children aged 8 years - autism and developmental disabilities monitoring network, 11 sites, United States, 2016. *MMWR Surveill. Summ.* 69 1–12. 10.15585/mmwr.ss6904a1 32214087 PMC7119644

[B31] Mars R. B. Neubert F. X. Noonan M. P. Sallet J. Toni I. Rushworth M. F. (2012). On the relationship between the “default mode network” and the “social brain”. *Front. Hum. Neurosci.* 6:189. 10.3389/fnhum.2012.00189 22737119 PMC3380415

[B32] Menon V. (2011). Large-scale brain networks and psychopathology: a unifying triple network model. *Trends Cogn. Sci.* 15 483–506. 10.1016/j.tics.2011.08.003 21908230

[B33] Molnar-Szakacs I. Uddin L. Q. (2013). Self-processing and the default mode network: interactions with the mirror neuron system. *Front. Hum. Neurosci.* 7:571. 10.3389/fnhum.2013.00571 24062671 PMC3769892

[B34] Muller R. A. Fishman I. (2018). Brain connectivity and neuroimaging of social networks in autism. *Trends Cogn. Sci.* 22 1103–1116. 10.1016/j.tics.2018.09.008 30391214 PMC7080636

[B35] Nielsen J. A. Zielinski B. A. Fletcher P. T. Alexander A. L. Lange N. Bigler E. D. (2014). Abnormal lateralization of functional connectivity between language and default mode regions in autism. *Mol. Autism.* 5:8. 10.1186/2040-2392-5-8 24502324 PMC3922424

[B36] Nomi J. S. Uddin L. Q. (2015). Developmental changes in large-scale network connectivity in autism. *Neuroimage Clin.* 7 732–741. 10.1016/j.nicl.2015.02.024 25844325 PMC4375789

[B37] Nordahl C. W. Mello M. Shen A. M. Shen M. D. Vismara L. A. Li D. (2016). Methods for acquiring MRI data in children with autism spectrum disorder and intellectual impairment without the use of sedation. *J. Neurodev. Disord.* 8:20. 10.1186/s11689-016-9154-9 27158271 PMC4858915

[B38] Padmanabhan A. Lynch C. J. Schaer M. Menon V. (2017). The default mode network in autism. *Biol. Psychiatry Cogn. Neurosci. Neuroimaging* 2 476–486. 10.1016/j.bpsc.2017.04.004 29034353 PMC5635856

[B39] Pelphrey K. A. Shultz S. Hudac C. M. Vander Wyk B. C. (2011). Research review: Constraining heterogeneity: the social brain and its development in autism spectrum disorder. *J. Child Psychol. Psychiatry* 52 631–644. 10.1111/j.1469-7610.2010.02349.x 21244421 PMC3096715

[B40] Pizzarelli R. Cherubini E. (2011). Alterations of GABAergic signaling in autism spectrum disorders. *Neural. Plast* 2011:297153. 10.1155/2011/297153 21766041 PMC3134996

[B41] Prat C. S. Stocco A. Neuhaus E. Kleinhans N. M. (2016). Basal ganglia impairments in autism spectrum disorder are related to abnormal signal gating to prefrontal cortex. *Neuropsychologia* 91 268–281. 10.1016/j.neuropsychologia.2016.08.007 27542318 PMC6453580

[B42] Reinders N. J. Branco A. Wright K. Fletcher P. C. Bryden P. J. (2019). Scoping review: physical activity and social functioning in young people with autism spectrum disorder. *Front. Psychol.* 10:120. 10.3389/fpsyg.2019.00120 30814964 PMC6381857

[B43] Roane H. S. Fisher W. W. Carr J. E. (2016). Applied behavior analysis as treatment for autism spectrum disorder. *J. Pediatr.* 175 27–32. 10.1016/j.jpeds.2016.04.023 27179552

[B44] Rubenstein J. L. Merzenich M. M. (2003). Model of autism: increased ratio of excitation/inhibition in key neural systems. *Genes Brain Behav.* 2 255–267. 10.1034/j.1601-183x.2003.00037.x 14606691 PMC6748642

[B45] Schopler E. Reichler R. J. DeVellis R. F. Daly K. (1980). Toward objective classification of childhood autism: Childhood Autism Rating Scale (CARS). *J. Autism Dev. Disord.* 10 91–103. 10.1007/BF02408436 6927682

[B46] Smith J. C. Nielson K. A. Antuono P. Lyons J. A. Hanson R. J. Butts A. M. (2013). Semantic memory functional MRI and cognitive function after exercise intervention in mild cognitive impairment. *J. Alzheimers Dis.* 37 197–215. 10.3233/JAD-130467 23803298 PMC4643948

[B47] Smith J. C. Paulson E. S. Cook D. B. Verber M. D. Tian Q. (2010). Detecting changes in human cerebral blood flow after acute exercise using arterial spin labeling: implications for fMRI. *J. Neurosci. Methods* 191 258–262. 10.1016/j.jneumeth.2010.06.028 20603148

[B48] Tager-Flusberg H. (2010). The origins of social impairments in autism spectrum disorder: studies of infants at risk. *Neural. Netw.* 23 1072–1076. 10.1016/j.neunet.2010.07.008 20800990 PMC2956843

[B49] Tomasi D. Volkow N. D. (2012). Resting functional connectivity of language networks: characterization and reproducibility. *Mol. Psychiatry* 17 841–854. 10.1038/mp.2011.177 22212597 PMC3323720

[B50] Towle P. O. Patrick P. A. Ridgard T. Pham S. Marrus J. (2020). Is earlier better? the relationship between age when starting early intervention and outcomes for children with autism spectrum disorder: a selective review. *Autism Res. Treat.* 2020:7605876. 10.1155/2020/7605876 32832154 PMC7421097

[B51] Tse A. C. Y. (2020). Brief report: impact of a physical exercise intervention on emotion regulation and behavioral functioning in children with autism spectrum disorder. *J. Autism Dev. Disord.* 50 4191–4198. 10.1007/s10803-020-04418-2 32130593

[B52] Uddin L. Q. Supekar K. Lynch C. J. Cheng K. M. Odriozola P. Barth M. E. (2015). Brain state differentiation and behavioral inflexibility in autism. *Cereb. Cortex* 25 4740–4747. 10.1093/cercor/bhu161 25073720 PMC4635916

[B53] Uddin L. Q. Supekar K. Menon V. (2013). Reconceptualizing functional brain connectivity in autism from a developmental perspective. *Front. Hum. Neurosci.* 7:458. 10.3389/fnhum.2013.00458 23966925 PMC3735986

[B54] Vander Wyk B. C. Hoffman F. Pelphrey K. A. (2014). Equivalent neural responses in children and adolescents with and without autism during judgments of affect. *Dev. Cogn. Neurosci.* 8 121–130. 10.1016/j.dcn.2013.08.001 24016745 PMC3931746

[B55] Vattikuti S. Chow C. C. (2010). A computational model for cerebral cortical dysfunction in autism spectrum disorders. *Biol. Psychiatry* 67 672–678. 10.1016/j.biopsych.2009.09.008 19880095 PMC3104404

[B56] Venkataraman A. Duncan J. S. Yang D. Y. Pelphrey K. A. (2015). An unbiased Bayesian approach to functional connectomics implicates social-communication networks in autism. *Neuroimage Clin.* 8 356–366. 10.1016/j.nicl.2015.04.021 26106561 PMC4474177

[B57] Webb J. T. Ferguson M. A. Nielsen J. A. Anderson J. S. (2013). BOLD Granger causality reflects vascular anatomy. *PLoS One* 8:e84279. 10.1371/journal.pone.0084279 24349569 PMC3862772

[B58] White S. J. Frith U. Rellecke J. Al-Noor Z. Gilbert S. J. (2014). Autistic adolescents show atypical activation of the brain’s mentalizing system even without a prior history of mentalizing problems. *Neuropsychologia* 56 17–25. 10.1016/j.neuropsychologia.2013.12.013 24361475

[B59] Xiao J. Chen H. Shan X. He C. Li Y. Guo X. (2021). Linked social-communication dimensions and connectivity in functional brain networks in autism spectrum disorder. *Cereb. Cortex* 31 3899–3910. 10.1093/cercor/bhab057 33791779 PMC8258445

[B60] Yerys B. E. Gordon E. M. Abrams D. N. Satterthwaite T. D. Weinblatt R. Jankowski K. F. (2015). Default mode network segregation and social deficits in autism spectrum disorder: Evidence from non-medicated children. *Neuroimage Clin.* 9 223–232. 10.1016/j.nicl.2015.07.018 26484047 PMC4573091

[B61] Yizhar O. Fenno L. E. Prigge M. Schneider F. Davidson T. J. O’Shea D. J. (2011). Neocortical excitation/inhibition balance in information processing and social dysfunction. *Nature* 477 171–178. 10.1038/nature10360 21796121 PMC4155501

[B62] Zeidman P. Jafarian A. Seghier M. L. Litvak V. Cagnan H. Price C. J. (2019). A guide to group effective connectivity analysis, part 2: Second level analysis with PEB. *Neuroimage* 200 12–25. 10.1016/j.neuroimage.2019.06.032 31226492 PMC6711451

[B63] Zhang L. Huang C. C. Dai Y. Luo Q. Ji Y. Wang K. (2020). Symptom improvement in children with autism spectrum disorder following bumetanide administration is associated with decreased GABA/glutamate ratios. *Transl. Psychiatry* 10:9. 10.1038/s41398-020-0692-2 32066666 PMC7026137

[B64] Zhao T. Liao X. Fonov V. S. Wang Q. Men W. Wang Y. (2019). Unbiased age-specific structural brain atlases for Chinese pediatric population. *Neuroimage* 189 55–70. 10.1016/j.neuroimage.2019.01.006 30625395

[B65] Zhou Y. Friston K. J. Zeidman P. Chen J. Li S. Razi A. (2018). The hierarchical organization of the default, dorsal attention and salience networks in adolescents and young adults. *Cereb. Cortex* 28 726–737. 10.1093/cercor/bhx307 29161362 PMC5929108

