# Supplementary material for: Alteration of Effective Connectivity in the Default Mode Network of Autism After an Intervention
Source: Front Neurosci. 2021 Dec 22;15:796437. doi: 10.3389/fnins.2021.796437 (PMC8727456; doi:10.3389/fnins.2021.796437)
Supplement: Supplementary file 1 [file Table_1.DOCX]

**Supplemental Table S1**

**Table S1** **Mini-basketball training content and goal for each course.**

| **Stages** | **Content** | **Goal** | **Duration (min)** |
| --- | --- | --- | --- |
| **classroom routine preparation** | Line up, classroom greetings, roll call, etc. | Social communication and interaction | 2 |
| **warm-up activities** | Stretching, jogging, limbs exercise, etc. | Warm-up | 8 |
| **mini-basketball training program** | Phase I: simple basketball training  Phase II: mini-basketball skill learning  Phase III: game based on mini-basketball | Social interaction and  mini-basketball skills  development | 25 |
| **cool-down activities** | Relaxation exercise and summary | Review, summary, reward, and clean-up | 5 |

The 12W-MBTP contents (40 min × 5 sessions per week × 12 weeks, fixed time, location, and physical educators) can be simply summarized as a three-phase program, with four small stages for each course including first 2 min classroom routine preparation, then 8 min warm-up activities, followed by a 25 min mini-basketball training program, and finally 5 min cool-down activities.
