# Supplementary material for: Alteration of Effective Connectivity in the Default Mode Network of Autism After an Intervention
Source: Front Neurosci. 2021 Dec 22;15:796437. doi: 10.3389/fnins.2021.796437 (PMC8727456; doi:10.3389/fnins.2021.796437)
Supplement: Supplementary file 2 [file Table_2.DOCX]

**Supplemental Table S2**

**Table S2** **Mini-basketball training program protocol.**

| **Phase** | **Goal** | **Content** | **Duration (week)** |
| --- | --- | --- | --- |
| Phase I | Standardize classroom  Routines and increase children’s interest in mini-basketball | Classroom routines (line up, classroom greetings, roll call, etc.) and simple basketball training (roll and throw the ball, etc.) | 2 |
| Phase II | Improve children's mini-basketball skills and social communication skills | Basic basketball skill (dribbling, passing, shooting, etc.)  Peer coordination training (passing and catching ball, relay racing, etc.) | 8 |
| Phase III | Improve children’s  cooperative ability, social  skills, and collectivization | Group game based on mini-basketball  (basketball-dribbling relay, basketball-passing relays, basket-moving shooting, playing ducks, etc.) | 2 |
