# Supplementary material for: Alteration of Effective Connectivity in the Default Mode Network of Autism After an Intervention
Source: Front Neurosci. 2021 Dec 22;15:796437. doi: 10.3389/fnins.2021.796437 (PMC8727456; doi:10.3389/fnins.2021.796437)
Supplement: Supplementary file 3 [file Table_3.DOCX]

**Supplemental Table S3**

**Table S3 the specific connection strength before and after the intervention of DCM**

|  | **Baseline** | | | **Posttest** | | |
| --- | --- | --- | --- | --- | --- | --- |
| HC_sub_001 | mPFC->mPFC | PCC->RTPJ | LTPJ->PCC | mPFC->mPFC | PCC->RTPJ | LTPJ->PCC |
| HC_sub_002 | 0.015229217 | -0.029572 | 0.31230398 | -0.25369495 | 0.17298117 | 0.04712673 |
| HC_sub_003 | 0.299113894 | 0.08656063 | -0.3218243 | -0.20629327 | 0.02542811 | 0.15094248 |
| HC_sub_004 | 0.190564986 | 0.01416205 | 0.33419579 | -0.66101764 | 0.2086934 | -0.0947076 |
| HC_sub_005 | -0.54862634 | 0.10322473 | -0.85514 | -0.46577673 | 0.04289193 | 0.0205071 |
| HC_sub_006 | 0.324560766 | 0.16087286 | 0.06942152 | -0.10209649 | 0.13963451 | 0.44345519 |
| HC_sub_007 | -0.07691806 | 0.05509397 | -0.1653452 | 0.371369575 | 0.10362473 | -0.1697384 |
| HC_sub_008 | 0.626709527 | -0.100075 | 0.06450942 | -0.10890435 | -0.191665 | 0.38427298 |
| HC_sub_009 | 0.447489169 | 0.07262707 | 0.27753902 | 0.421753211 | 0.04605754 | 0.18277643 |
| HC_sub_010 | -0.10316868 | 0.39421508 | 0.00342667 | -0.46878383 | -0.0860486 | 0.12645325 |
| HC_sub_011 | 0.328380679 | -0.2439844 | -0.2719399 | -0.58408903 | 0.07180965 | -0.2377825 |
| HC_sub_012 | -0.11948559 | -0.0941133 | 0.09003026 | -0.51573268 | 0.0050273 | 0.01415855 |
| HC_sub_013 | -1.27243822 | 0.03920716 | 0.16679568 | 0.688850775 | 0.19817509 | 0.04900289 |
| HC_sub_014 | 0.189533024 | 0.13661322 | 0.52241045 | -0.05831926 | -0.0256958 | 0.06474865 |
| PT_sub_001 | -0.12385805 | 0.21547092 | -0.6123524 | -0.70424214 | 0.14630464 | -0.2500775 |
| PT_sub_002 | -0.2556381 | 0.12747323 | 0.588088 | 0.475174217 | 0.06012654 | -0.1760884 |
| PT_sub_003 | 0.113732648 | 0.12439903 | 0.11860683 | 0.90585463 | 0.20755048 | -0.0321974 |
| PT_sub_004 | -0.32594105 | 0.06228811 | -0.8384711 | -0.01982718 | -0.0161225 | -0.220167 |
| PT_sub_005 | -0.51879778 | 0.14167922 | -0.6904804 | -0.23576458 | 0.32125023 | 0.03849347 |
| PT_sub_006 | -0.25520452 | 0.10677254 | 0.78931278 | 0.112547889 | 0.07689067 | -0.1939402 |
| PT_sub_007 | 0.139692367 | 0.01255044 | 0.36531003 | 0.347896429 | 0.03764122 | 0.03721981 |
| PT_sub_008 | 0.540641693 | 0.29251804 | 0.36907777 | -0.13644223 | -0.2043735 | -0.6099627 |
| PT_sub_009 | 0.673736793 | 0.29837446 | -0.0427915 | -0.02727951 | -0.0944134 | -0.1100249 |
| PT_sub_010 | 0.335338678 | 0.19057186 | -0.2565938 | 0.159209855 | 0.2781715 | 0.05990714 |
| PT_sub_011 | 0.151685767 | -0.288017 | 0.15306417 | 0.07980642 | -0.0268156 | -0.0711229 |
| PT_sub_012 | -0.14910895 | 0.29899527 | 0.55498542 | 0.573144773 | -1.1314657 | 0.36777978 |
| PT_sub_013 | 0.192242541 | 0.3069058 | 0.03233 | 0.072001337 | -0.1609672 | 0.53274647 |
| PT_sub_014 | 0.417140804 | 0.30947782 | -0.2091765 | 0.219499586 | 0.01526908 | -0.0977836 |
| PT_sub_015 | 0.124706661 | 0.00932732 | 0.12221733 | 0.193816968 | -0.145721 | 0.23966936 |
| PT_sub_016 | -0.25557914 | 0.17060155 | 0.16469184 | 0.349848222 | 0.05434693 | -0.0634604 |
| PT_sub_017 | -0.53233583 | -0.44175 | -0.0002543 | 0.292141206 | -0.0296481 | -0.0067681 |
| HC_sub_001 | 0.511251626 | 0.07201862 | 0.17360716 | 0.704899046 | 0.27109572 | 0.05003247 |
