# Supplementary figures and images for: Alteration of Effective Connectivity in the Default Mode Network of Autism After an Intervention
Source: Front Neurosci. 2021 Dec 22;15:796437. doi: 10.3389/fnins.2021.796437 (PMC8727456; doi:10.3389/fnins.2021.796437)

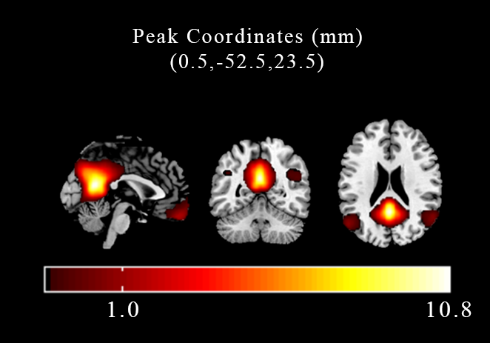

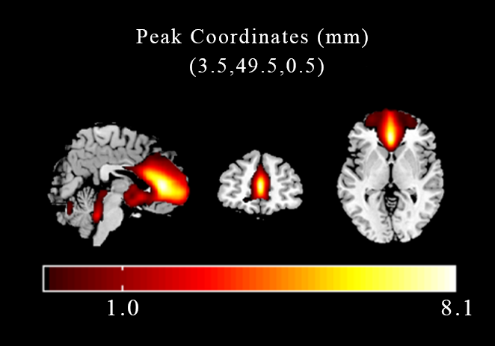

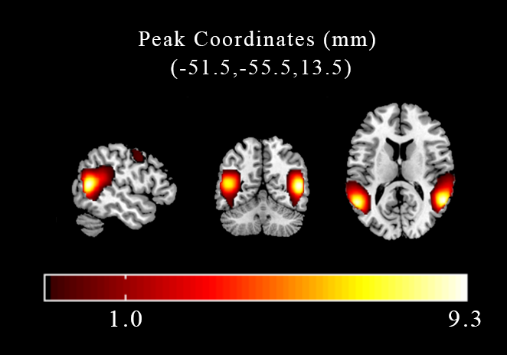

Supplement: Supplementary file 4 [file Table_4.DOCX]
