# Supplementary material for: Alteration of Effective Connectivity in the Default Mode Network of Autism After an Intervention
Source: Front Neurosci. 2021 Dec 22;15:796437. doi: 10.3389/fnins.2021.796437 (PMC8727456; doi:10.3389/fnins.2021.796437)
Supplement: Supplementary file 5 [file Data_Sheet_1.DOCX]

**Supplementary Materials**

**Follow-up simple effect analysis results for the subscales of SRS-2 are presented below:** (1) the pre-test social cognition score was significantly higher than that at post-test in the experimental group (*F* _(1, 29)_ = 7.206, p = 0.012), where no significant change from the post to pre-test was observed in the control group; (2) the post-test social communication score was significantly lower than that at baseline in the experimental group (*F* _(1, 29)_ = 4.715, p = 0.038), whereas a higher score was observed in the control group from the post-test to pre-test (*F* _(1, 29)_ = 4.839, p = 0.036; higher scores indicate severe symptoms); (3) there was no significant difference between baseline and post-test in the experimental group (*F* _(1, 29)_ = 0.659, p > 0.05) in terms of autistic mannerisms, whereas the post-test score was significantly higher than that at baseline in the control group (*F* _(1, 29)_ = 5.202, p = 0.03).
